# Molecular evolution of bumble bee vitellogenin and vitellogenin‐like genes

**DOI:** 10.1002/ece3.7736

**Published:** 2021-06-05

**Authors:** Fang Zhao, Claire Morandin, Kai Jiang, Tianjuan Su, Bo He, Gonghua Lin, Zuhao Huang

**Affiliations:** ^1^ School of Life Sciences Jinggangshan University Ji’an China; ^2^ Department of Ecology and Evolution, Biophore University of Lausanne Lausanne Switzerland

**Keywords:** vitellogenin, *Bombus*, positive selection, functional pleiotropy

## Abstract

Vitellogenin (Vg), a storage protein, has been significantly studied for its egg yolk precursor role in oviparous animals. Recent studies found that vitellogenin and its Vg‐like homologs were fundamentally involved in many other biological processes in social insects such as female caste differences and oxidative stress resilience. In this study, we conducted the first large‐scale molecular evolutionary analyses of vitellogenin coding genes (*Vg*) and *Vg‐like* genes of bumble bees, a primitively eusocial insect belonging to the genus Bombus. We obtained sequences for each of the four genes (*Vg*, *Vg‐like‐A*, *Vg‐like‐B*, and *Vg‐like‐C*) from 27 bumble bee genomes (nine were newly sequenced in this study), and sequences from the two closest clades of *Bombus*, including five *Apis* species and five *Tetragonula species*. Our molecular evolutionary analyses show that in bumble bee, the conventional *Vg* experienced strong positive selection, while the *Vg‐like* genes showed overall relaxation of purifying selection. In *Apis* and *Tetragonula;* however, all four genes were found under purifying selection. Furthermore, the conventional *Vg* showed signs of strong positive selection in most subgenera in *Bombus*, apart from the obligate parasitic subgenus *Psithyrus* which has no caste differentiation. Together, these results indicate that the conventional *Vg*, a key pleiotropic gene in social insects, is the most rapidly evolving copy, potentially due to its multiple known social functions for both worker and queen castes. This study shows that concerted evolution and purifying selection shaped the evolution of the *Vg* gene family following their ancient gene duplication and may be the leading forces behind the evolution of new potential protein function enabling functional social pleiotropy.

## Graphical abstract

In this study, we conducted the first large‐scale molecular evolutionary analyses of vitellogenin‐coding genes (*Vg*) and *Vg*‐like genes (Vg‐like‐A, Vg‐like‐B, and Vg‐like‐C) of bumble bees, a primitively eusocial insects belonging to the genus Bombus. The overall dN/dS ratio of the conventional Vg was 1.311, and this gene was under strong positive selection in most (9/10) subgenera in Bombus, apart from the obligate parasitic subgenus Psithyrus (dN/dS = 0.713) which has no caste differentiation.

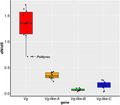

## 1 INTRODUCTION

Vitellogenin (Vg) is a phospholipoglycoprotein, and the precursor protein of vitellin, a protein required for egg yolk formation by most oviparous species (Spieth et al., 1991). In insects, vitellogenin is synthesized in the fat body, released into the hemolymph, and finally taken up by developing oocytes to be consumed throughout embryogenesis (Hagedorn & Kunkel, 1979; Pan et al., 1969; Raikhel & Dhadialla, 1992; Tufail & Takeda, 2008). Despite its main egg yolk function, Vg is not female‐specific and can also be found in males of some species although in smaller amount (Piulachs et al., 2003; Trenczek & Engels, 1986; Tufail & Takeda, 2008). Moreover, Vg has been extensively studied for its multifunctional effects in social insect life histories. In honey bee (*Apis mellifera*), in addition to its central involvement in the division of labor between queens and workers (Tufail & Takeda, 2008; Weil et al., 2009), Vg is known to be involved in regulation of nonreproductive features of colonies, such as aging and queen longevity (Corona et al., 2007; Excels, 1974), temporal worker division of labor (Bloch & Grozinger, 2011; Guidugli et al., 2005; Münch & Amdam, 2010; Nelson et al., 2007), and royal jelly production (Amdam et al., 2003).

In honey bee, only one conventional Vg gene can be found and has been extensively studied for its multiple phenotypic effects on both queen and worker traits (Amdam et al., 2003). Pleiotropic genes are expected to be evolutionarily constrained since mutations that increase fitness for one trait might decrease overall fitness via antagonistic effects on other traits (Otto, 2004). Interestingly however, many previous studies have not found support for a negative relationship between the pleiotropy of a given gene and its selection pressure, measured as the ratio of nonsynonymous to synonymous substitutions (*dN/dS*; e.g., Razeto‐Barry et al., 2011; Twyman et al. 2018; Vedanayagam & Garrigan, 2015). *Vg* displayed high rates of adaptive evolution, and positive selection signs of this gene were repeatedly detected in eusocial hymenopteran species (Kent et al., 2011; Morandin et al., 2014; Salmela et al., 2016).

Multiple roles for a single protein are also projected to lead to a gene duplication event and to favor the multiple acquired roles. Besides the conventional Vg, three homologs called Vg‐like proteins were recently discovered in ants (Morandin et al., 2014). These Vg homologs have arisen from an ancient gene duplication event. Two of these homologs, Vg‐like‐A and Vg‐like‐B, can be found in all insect species studied, while Vg‐like‐C was so far only found in Hymenoptera (Kohlmeier et al., 2018; Morandin et al., 2014). These homologs exhibit differences in their conserved protein domains and have undergone rapid evolution after duplications (Morandin et al., 2014). Their role is currently unknown, but their structural variation suggests variable functions. In honey bee, Vg‐like‐A displays the closest structural and functional similarities to Vg and responded strongly to inflammatory and oxidative conditions, thus is likely associated with the aging process (Salmela et al., 2016). Vg‐like‐A also showed a strong temporal expression variation and may be involved in wintering worker longevity (Ricigliano et al., 2018). Furthermore, Vg‐like‐A is linked to the regulation of nursing behaviors in the ant *Temnothorax longispinosus* (Kohlmeier et al., 2018). During duplication, Vg‐like‐B lost several Vg structural elements, which may suggest that Vg‐like‐B may perform only few of the Vg original functions, such as coping with oxidative stress (Morandin et al., 2014). Four protein domains (N‐sheet, a‐helical, vWFD, and polyserine linker) were found in Vg, while only the N‐sheet was detected in Vg‐like‐C, potentially implying specialization, and could possibly be involved in neurobiological functions (Salmela et al., 2016).

Bumble bees are a group of insects belonging to the genus *Bombus* (Hymenoptera: Apidae). Bumble bees, honey bees, and stingless bees (*Tetragonula*) are phylogenetically close relatives (Peters et al., 2017). There are about 250 known bumble bee species belonging to 15 subgenera, mainly distributed in the northern hemisphere (Cameron et al., 2007; Williams et al., 2008). Bumble bees are often described as primitively eusocial because their social organization is simpler than that of the honeybee. Unlike honey bees or stingless bees, most bumble bee species have an annual cycle, with queens single‐handedly founding nests (Goulson, 2010). Bumble bees pass through several distinct phases during their annual life cycle, including solitary and eusocial phases. At the final stage of their colony cycle, termed the competition phase, the queen and workers will compete intensely over the production of males (Amsalem et al., 2015).

The fascinating life history and high levels of biological and ecological heterogeneity make bumble bees an outstanding model system for the study of molecular evolution. First of all, the biological and ecological characteristics of bumble bees can largely differ among the different subgenera (Williams et al., 2008). For instance, the tongue lengths of bumble bees are very diversified among different subgenera. Some subgenera such as *Orientalibombus*, *Subterraneobombus*, and *Sibiricobombus* which favor deep flowers have very long tongues, while others have relatively short tongues (Williams et al., 2008). Bumble bees are also extremely diversified in their habitats: for example, *Mendacibombus* and *Alpinobombus* species prefer alpine/arctic, while *Orientalibombus* generally use forest habitats. Most strikingly, there are obligate parasitic species in the subgenus *Psithyrus* which enslaves the species from other subgenera (Amsalem et al., 2015). To our knowledge, such extreme biological and ecological diversifications within a single genus have not been previously reported in honey bees or stingless bees. Also, honey bees and stingless bees are primarily tropical insects, with relatively stable environments, while bumble bees mainly occur in cool climates in general with more variable environments. Furthermore, bumble bees biological and ecological characteristics may also deviate among different species within a single subgenus. For example, in at least seven subgenera, the distribution elevation significantly varies among different species (An et al., 2014), leading to distinct genomic evolution rates among species (Lin et al., 2019). Consequently, we hypothesize that Vg and Vg‐like genes should be under distinct selection forces in bumble bees compared to honey bees or stingless bees.

To date, for most multi‐species Vg analysis, bumble bee *Vg* sequences were compared with species from other genera (Du et al., 2019; Li et al., 2010; Salmela et al., 2016), probably due to the lack of sequence data from multiple bumble bee species. In this study, based on 27 bumble bee genomes, and combined with genomes of their phylogenetically close relatives (honey bees and stingless bees), we conducted the first large‐scale molecular evolutionary analyses of bumble bee *Vg* and *Vg‐like* genes to understand the selective patterns of Vg gene family in bumble bees.

## 2 MATERIALS AND METHODS

### 2.1 DNA extraction and libraries construction

Adult workers from nine species of *Bombus* (*B*. *lantschouensis*, *B*. *sichelii*, *B*. *impetuosus*, *B*. *laesus*, *B*. *skorikovi*, *B*. *bohemicus*, *B*. *trifasciatus*, *B*. *waltoni*, and *B*. *convexus*) were live‐trapped using sweep nets from the field. Samples of *B*. *trifasciatus* were collected in Ji'an, Jiangxi Province, China, while the other eight species were collected in Qinghai Province, China, between 2017 and 2019. After collection, samples were immediately stored at −20°C.

DNA extractions, libraries construction, and sequencing were done by the company Novogene. In brief, DNA was extracted from whole‐body of each individual (one worker per species) using a DNeasy Blood and Tissue Kit (Qiagen). Concentration and quality of extracted DNA was examined with an Agilent 2100 Bioanalyzer (Agilent Technologies). Libraries were sequenced paired‐end for 150 cycles (1 × 150 bp) on an Illumina Hiseq 2000 system. Read quality was inspected with FASTX‐Toolkit (http://hannonlab.cshl.edu/fastx_toolkit/). Clean reads were used for de novo assembly using IDBA‐UD (Peng et al., 2012) with default settings, and contigs shorter than 200 bp were discarded.

### 2.2 Discovery of *Vg* and *Vg‐like* sequences

**FIGURE 1:**
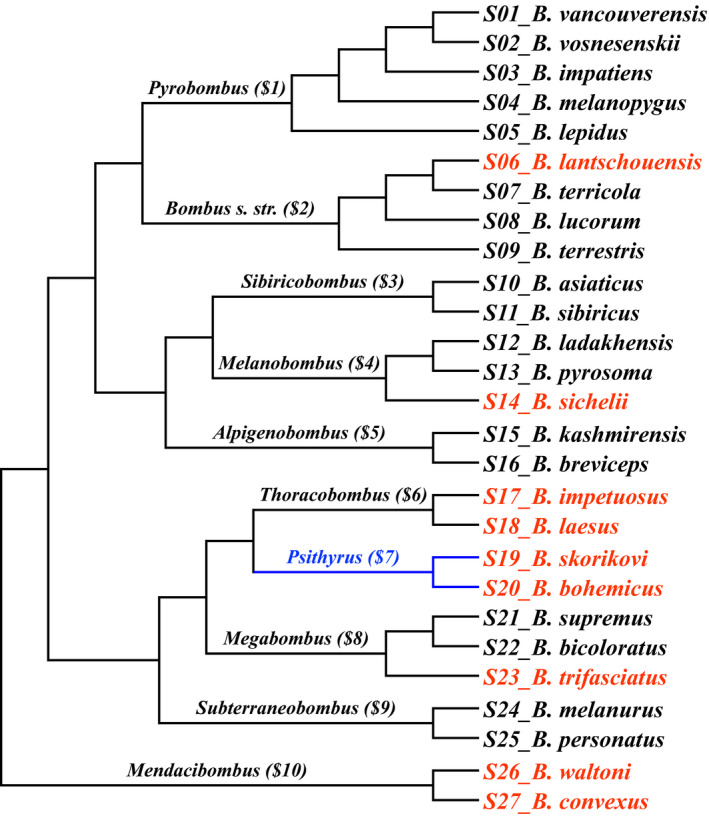
Phylogenetic relationships of the 27 bumble bee species involved in this study (red color, species whose genomes were sequenced in this study; blue color, obligate parasitic subgenus)

In addition to our nine newly sequenced *Bombus* species, previously published bumble bee genomes were added to our dataset (see Jackson et al., 2020; Kent et al., 2018; Lin et al., 2019; Sadd et al., 2015; Tian et al., 2019). As a result, a total of 27 bumble bee species belonging to 10 subgenera (2 to 5 species for each genus) were used for this study (Figure 1; Table S1). The nucleotide sequences of *Vg*, *Vg‐like‐A*, *Vg‐like‐B*, and *Vg‐like‐C* of *B. impatiens* were downloaded from GenBank (*Vg*, XP_003492277.1; *Vg‐like‐A*, XP_003494500.3; *Vg‐like‐B*, XP_012245829.1; and *Vg‐like‐C*, XP_003489777.1). We retrieved the corresponding coding sequences from the 26 other bumble bee genomes using the software Exonerate v2.4.0 (Slater & Birney, 2005), with default settings and with *B. impatiens* Vg and Vg‐like protein sequences as queries.

Additionally, we aimed to retrieve *Vg* and *Vg‐like* sequences from stingless bees (*Tetragonula* spp.) and honey bees (*Apis* spp.). These two genera are phylogenetically close relatives of *Bombus* (Peters et al., 2017) and five species of each genus (*Tetragonula carbonaria*, *T*. *clypearis*, *T*. *davenporti*, *T*. *hockingsi*, and *T*. *mellipes*; *Apis cerana*, *A*. *dorsata*, *A*. *florea*, *A*. *laboriosa*, and *A*. *mellifera*) were available with genomic resources in GenBank (Table S2). To do so, we obtained *Vg* and *Vg‐like* sequences from GenBank for *Apis mellifera* (*Vg*, NP_001011578; *Vg‐like‐A*, XP_001121939; *Vg‐like‐B*, XP_395423; *Vg‐like‐C*, XP_001122505) and used them as queries to extract the other *Apis* species sequences using Exonerate with default settings. In the same manner, the translated protein sequences of the four genes from *B. impatiens* and *A*. *mellifera* were used as queries to extract the corresponding sequences from the five *Tetragonula* species.

The *Vg* and *Vg‐like* sequences from each species were aligned for each genus separately using ClustalW (Codons) program embedded in the software MEGA v10.1.7 (Kumar et al., 2018) with default settings and verified by visual inspection. The DNA sequence variations were calculated using DnaSP v 6.12.03 (Rozas et al., 2017).

The reference species tree of *Bombus* (Figure 1) was drawn by TreeGraph2 v2.15.0 (Stöver & Müller, 2010) according to previous studies (Cameron et al., 2007; Williams et al., 2008, 2012). The reference species tree of *Tetragonula* (Figure S1) was drawn according to Rasmussen and Cameron (2010) and Hereward et al. (2020). The reference tree of *Apis* was drawn according to Raffiudin and Crozier (2007; Figure S1).

### 2.3 Molecular evolutionary analyses among different genera

We used the CODEML program in the PAML package v4.9j (Yang, 2007) to study the selection pressures affecting the different genes and to test for patterns of molecular evolution. Our aim was to estimate synonymous and nonsynonymous substitutions using the branch tests and site tests (Bielawski & Yang, 2005).

We firstly tested whether the overall selection of *Vg* and *Vg‐like* genes of bumble bees deviated from honey bees and stingless bees. The M0 (one‐ratio) was used to estimate the overall selection (*dN/dS*, ratio of nonsynonymous / synonymous substitution rates) across all sites, and the alignment of each gene for each genus (*Bombus*, *Apis*, and *Tetragonula*) was separately used to calculate the *dN/dS* ratio of each genus. Next, we calculated the pairwise *dN/dS* ratios (Yang & Nielsen, 2000) across the species within each genus using the YN00 program from the PAML package. The values of pairwise *dN/dS* ratios of each gene were compared between *Bombus* and each of the other two genera using a Mann–Whitney rank test in SPSS v25.0. The relationships between *dN* and *dS* and between *dN/dS* and *dS* were tested using a linear regression analysis in SPSS.

### 2.4 Molecular evolutionary analyses within *Bombus*

We first tested whether differences of selection pressures exist among the ten bumble bee subgenera. The “several *dN/dS* ratios” branch model (BM) was used to estimate the *dN/dS* ratios of each of the ten subgenera independently. The ten external branches corresponding to the ten subgenera were viewed as different foregrounds, whereas all the internal branches were viewed as a common background (Figure 1). The likelihood ratio tests (LRTs) between M0 (null model) and BM models were conducted by comparing twice the difference in log‐likelihood values (*2ΔlnL*) against a chi‐square distribution (*df* = 2). The obtained ten *dN/dS* ratios of the bumble bee subgenera were compared across the four different genes using one‐way ANOVA and paired samples *t* tests in SPSS. Moreover, we also used the branch‐site model called Model A to test for positive selections in each subgenera. The null model for Model A is Model A1, which is a modify on Model A, but with *ω*2 = 1 fixed (Yang et al., 2005; Zhang et al., 2005). In each run, one target subgenus was marked as the foreground branch while the remaining nine subgenera were viewed as backgrounds. Again, *2ΔlnL* values between Model A and Model A1 were used to conduct LRTs for robustness with χ^2^ test (*df* = 1).

Lastly, we studied the extent of selection for each *Vg* and *Vg‐like* gene set of *Bombus* individually by dividing the data into four data sets (one for each orthologous gene) and comparing the neutral model (M1a) with a model allowing positive selection (M2a). The *2ΔlnL* values between the M1a and M2a models were used to test for robustness using LRTs with χ^2^ test (*df* = 2), and positively selected sites were identified with the Bayes Empirical Bayes (BEB; Yang et al. 2005).

It should be mentioned that, in order to reduce false discovery rate, a Benjamini–Hochberg correction in R program (“p.adjust” command) was used where necessary (see below).

## 3 RESULTS

### 3.1 Overall genetic variation of *Vg* and *Vg‐like* genes

A total of 2,095 million DNA reads were obtained from the nine bumble bee species (*B*. *lantschouensis*, *B*. *sichelii*, *B*. *impetuosus*, *B*. *laesus*, *B*. *skorikovi*, *B*. *bohemicus*, *B*. *trifasciatus*, *B*. *waltoni*, and *B*. *convexus*), with a total size of 314 Gb (SRA accession No. PRJNA667279). Coding sequences of the four Vg genes were successfully obtained for the 27 bumble bee species (File S1). The lengths of the aligned sequences (stop codons not considered) were 5,337, 4,569, 4,260, and 960 base pairs, respectively. *Vg* was the most variable in terms of nucleotide sequence identity among the four genes, with 2,389 (42.98%) variable nucleotide sites, including 39 indel codons. In contrast, *Vg‐like‐B* was the most conservative, with 11.56% variable nucleotide sites, and no indel was detected. *Vg‐like‐A* and *Vg‐like‐C* showed similar levels of genetic variation and *Vg‐like‐C* sequence included three indel codons (Table 1).

**TABLE 1:** Genetic variations of *Vg* and *Vg‐like* genes of the bumble bees (*Bombus*), stingless bees (*Tetragonula*), and honey bees (*Apis*; stop codons were not considered)

Sequence	Index	*Vg*	*Vg‐like‐A*	*Vg‐like‐B*	*Vg‐like‐C*
*Bombus* (*N* = 27)	Total sites	5,337	4,569	4,260	960
	Variable sites	2,294	903	476	193
	Variable percent	42.98%	19.76%	11.17%	20.10%
*Tetragonula* (*N* = 5)	Total sites	5,331	4,503	4,245	948
	Variable sites	140	73	54	39
	Variable percent	2.63%	1.62%	1.27%	4.11%
*Apis* (*N* = 5)	Total sites	5,319	4,524	4,260	978
	Variable sites	839	514	194	213
	Variable percent	15.77%	11.36%	4.55%	21.78%

The sequences of the four genes from five stingless bee species and five honey bee species are also provided in File S1. Unlike bumble bees, stingless bees and honey bees *Vg‐like‐C* sequence was the most variable in terms of nucleotide sequence identity among the four genes, follows by *Vg* and *Vg‐like‐A* (Table 1). Consistent with the bumble bee sequences, *Vg‐like‐B* was the most conserved gene for both stingless and honey bees (Table 1). The overall sequence variation information can be found in Table 1.

### 3.2 Molecular evolution between *Bombus* and the other two genera

We first characterized the extent of positive and purifying selection in each of the orthologous copies separately (*Vg*, *Vg‐like‐A*, *Vg‐like‐B*, and *Vg‐like‐C*). The *dN/dS* ratios based on the M0 model showed that *Bombus* conventional *Vg* was under strong positive selection (*dN/dS* = 1.311), whereas the *Vg‐like* genes were under purifying selection (*dN/dS* = 0.349, 0.077 and 0.196, respectively, Table 2). In *Apis* and *Tetragonula*, all four genes were found under purifying selection. Moreover, in *Tetragonula*, *Vg‐like‐A*, *B*, and *C* are the most conserved copies (d*N*/d*S* = 0.295, 0.072 and 0.107, respectively, Table 2), whereas the conventional *Vg* evolve more rapidly (*dN*/*dS* = 0.302). In *Apis*, however, *Vg‐like‐C* was evolving more rapidly (*dN/dS* = 0.301) than the conventional *Vg* (*dN/dS* = 0.260), *Vg‐like‐A* (*dN/dS* = 0.211), and *Vg‐like‐B* (*dN/dS* = 0.076). In all three genera, *Vg‐like‐B* was the most conserved gene (Table 2).

**TABLE 2:** The overall *dN/dS* ratio of *Vg* and *Vg‐like* genes based on M0 model

Genus	*Vg*	*Vg‐like‐A*	*Vg‐like‐B*	*Vg‐like‐C*
*Bombus* (*N* = 27)	1.311	0.349	0.077	0.196
*Tetragonula* (*N* = 5)	0.302	0.295	0.072	0.107
*Apis* (*N* = 5)	0.260	0.211	0.076	0.301

**FIGURE 2:**
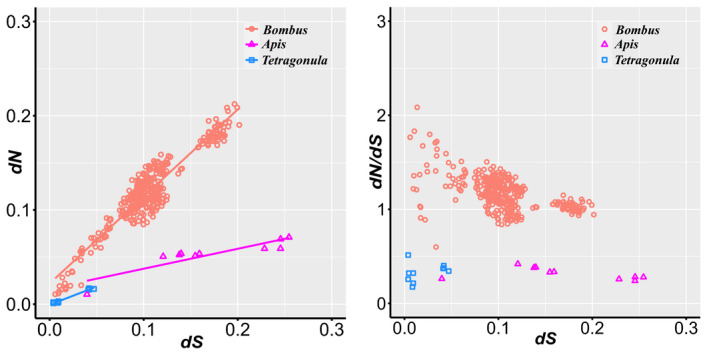
Scatter plot of *dN* versus *dS* (left) and *dN/dS* versus *dS* (right). The *dN*, *dS*, and *dN/dS* values were calculated under pairwise models among different species within genus *Bombus*, *Tetragonula*, and *Apis*, respectively

Pairwise analyses of *Vg* selection showed that, for any of the three genera, *dN* significantly increased with increasing *dS* (Figure 2). Linear regression and Benjamini–Hochberg correction (*n* = 12) showed that the relationship of *dN* and *dS* of *Bombus* species follow a function of *dN* = 0.022 + 0.924*dS, with a strong correlation (*R* ^2^ = 0.882, *p* < .001). Similar patterns were found among *Tetragonula* species (*dN* = −6.26E‐4 + 0.385 * *dS*, *R* ^2^ = 0.985, *p* < .001) and *Apis* species (*dN* = 0.016 + 0.212 * *dS*, *R* ^2^ = 0.788, *p* < .001). For all Vg orthologous copies, *dN* and *dS* were also positively correlated, at the exception of *Vg‐like‐C* sequence in *Apis* (*R* ^2^ = 0.281, *p* =.115, Table 3). The linear regression information is shown in Table 3 and Figure S2.

**TABLE 3:** Linear regression between pairwise *dN* and *dS* of among bumble bee species

Gene	Genus	Equation	Statistical index
*Vg*	*Bombus*	*dN* = 0.924*dS + 0.022	*R* ^2^ = .882, *p* < .001
	*Tetragonula*	*dN* = 0.385*dS − 6.26E−4	*R* ^2^ = .985, *p* < .001
	*Apis*	*dN* = 0.212*dS + 0.016	*R* ^2^ = .788, *p* < .001
*Vg‐like‐A*	*Bombus*	*dN* = 0.434*dS − 8.51E−4	*R* ^2^ = .914, *p* < .001
	*Tetragonula*	*dN* = 0.208*dS + 1.41E−3	*R* ^2^ = .979, *p* < .001
	*Apis*	*dN* = 0.212*dS + 1.74E−3	*R* ^2^ = .666, *p* =.004
*Vg‐like‐B*	*Bombus*	*dN* = 0.090*dS − 4.90E−4	*R* ^2^ = .709, *p* < .001
	*Tetragonula*	*dN* = 0.063*dS + 4.72E−4	*R* ^2^ = .970, *p* < .001
	*Apis*	*dN* = 0.078*dS + 1.13E−4	*R* ^2^ = .760, *p* =.001
*Vg‐like‐C*	*Bombus*	*dN* = 0.271*dS − 6.48E−3	*R* ^2^ = .856, *p* < .001
	*Tetragonula*	*dN* = 0.251*dS + 8.03E−4	*R* ^2^ = .977, *p* < .001
	*Apis*	*dN* = 0.129*dS + 0.057	*R* ^2^ = .281, *p* =.115

Mann–Whitney rank test and Benjamini–Hochberg correction (*n* = 8) showed that pairwise *dN/dS* values of bumble bees *Vg* were significantly larger than those of stingless bees and honey bees (*Z* = −5.393, *p* < .001). Pairwise *dN/dS* values of bumble bees *Vg‐like‐A* were significantly larger than those of honey bees (*Z* = −5.128, *p* < .001), but not significantly deviated from stingless bees (*Z* = −1.023, *p* =.350). No significant differences of pairwise *dN/dS* values for *Vg‐like‐B* could be detected between bumblebees and stingless bees (*Z* = −1.540, *p* =.165) nor honey bees (*Z* = −0.255, *p* =.799). In contrast, pairwise *dN/dS* values of bumble bees *Vg‐like‐C* were significantly smaller than those of stingless bees (*Z* = −3.306, *p* =.002) and honey bees (*Z* = −4.491, *p* < .001).

### 3.3 Molecular evolution within *Bombus*

**FIGURE 3:**
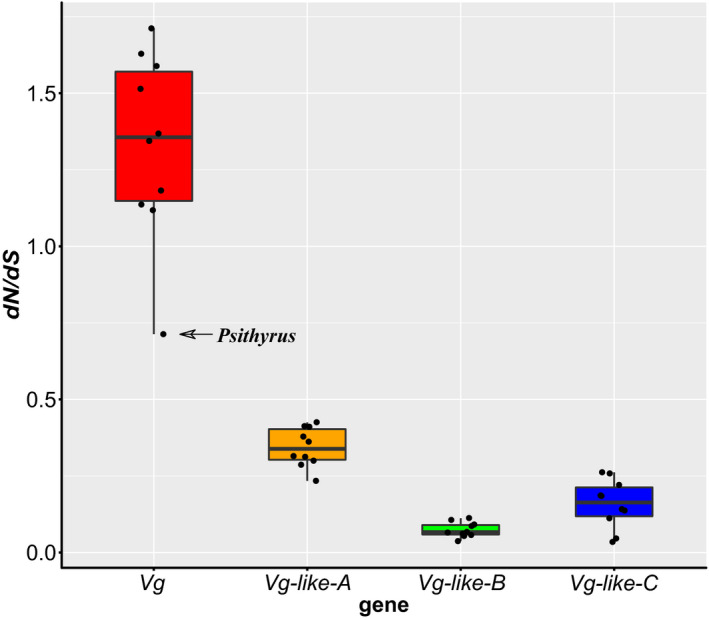
The *dN/dS* ratios of *Vg* and *Vg‐like* genes of bumble bees based on branch model

Similar as the M0 model, the branch model showed that *Bombus* conventional *Vg* was under positive selection with d*N/dS* = 1.331 ± 0.302 (mean ± *SD*), whereas the *Vg‐like* genes were under purifying selection (*dN/dS* = 0.344 ± 0.064, 0.074 ± 0.024 and 0.159 ± 0.080, respectively [Table 4; Figure 3]). One‐Way ANOVA based on the branch model results showed that the *dN/dS* values significantly deviated among the four genes (*F* = 131.588, *df* = 3, *p* < .001). Paired sample *t* test and Benjamini–Hochberg correction (*n* = 6) confirmed this finding (*df* = 9, *p* <= .006) and showed a rank order of *Vg > Vg‐like‐A > Vg‐like‐C > Vg‐like‐B* (Figure 3). According to the likelihood ratio test, the branch model significantly deviated from the M0 (*df* = 10, *p* < .001) for *Vg*, but not for the *Vg‐like* genes (*df* = 10, *p* >= .151; Table 4). For *Bombus Vg*, nine out of ten subgenera showed a *dN/dS* ratios over 1 (average = 1.40, range: 1.12–1.71, Table 4), with the exception of the subgenus *Psithyrus* (Figure 3) for which the *dN/dS* ratios was only 0.713.

**TABLE 4:** *dN/dS* ratios for 10 bumblebee subgenera based on branch model and LRTs between branch model and M0 (null model)

Subgenus	*Vg*	*Vg‐like‐A*	*Vg‐like‐B*	*Vg‐like‐C*
*Pyrobombus*	1.514	0.379	0.091	0.221
*Bombus* *s. str*.	1.118	0.413	0.086	0.112
*Sibiricobombus*	1.712	0.411	0.065	0.185
*Melanobombus*	1.628	0.312	0.062	0.046
*Alpigenobombus*	1.344	0.286	0.038	0.142
*Thoracobombus*	1.589	0.362	0.112	0.138
*Psithyrus*	0.713	0.300	0.068	0.262
*Megabombus*	1.368	0.315	0.054	0.187
*Subterraneobombus*	1.182	0.234	0.058	0.034
*Mendacibombus*	1.137	0.425	0.106	0.259
*2∆lnL*	33.518	9.330	11.658	14.514
*p* (*df* = 10)	<.001	.501	.309	.151

The M2a model showed that 32.1% of the *Bombus Vg* codons were under purifying selection (*dN/dS* < 1), 46.2% under neutral selection (*dN/dS* = 1), and 21.6% under positive selection (*ω*2 = 4.111). For the *Vg‐like* genes, most codons were under either purifying selection or neutral selection (Table 5). In *Bombus*, Bayes Empirical Bayes analysis showed that 150 codons (8.43%) in the conventional *Vg* and 3 codons (0.20%) in *Vg‐like‐A* were detected as positively selected sites (probability >.95). Yet, we could not detect any positively selected sites for *Vg‐like‐B* and *Vg‐like‐C*. The M2a models were robust for *Vg* and *Vg‐like‐A*, but not for *Vg‐like‐B* or *Vg‐like‐C* (Table S3).

**TABLE 5:** Statistical results of site models in *Vg* and *Vg‐like* genes of 27 bumble bee species

Gene	M1a (np = 54)	M2a (np = 56)	LRT (*df* = 2)
*Vg*	*p*0 = 0.436, *ω*0 = 0.083; *p*1 = 0.564, *ω*1 = 1.000; *lnL* = −31,891.635	*p*0 = 0.321, *ω*0 = 0.070; *p*1 = 0.462, *ω*1 = 1.000; *p*2 = 0.216, *ω*2 = 4.111; *lnL* = −31,416.198	*2∆lnL* = 950.874, *p* < .001
*Vg‐like‐A*	*p*0 = 0.716, *ω*0 = 0.064; *p*1 = 0.284, *ω*1 = 1.000; *lnL* = −13,541.057	*p*0 = 0.746, *ω*0 = 0.091; *p*1 = 0.231, *ω*1 = 1.000; *p*2 = 0.023, *ω*2 = 3.917; *lnL* = −13,526.160	*2∆lnL* = 29.794, *p* < .001
*Vg‐like‐B*	*p*0 = 0.968, *ω*0 = 0.050; *p*1 = 0.032, *ω*1 = 1.000; *lnL* = −9,062.035	*p*0 = 0.971, *ω*0 = 0.052; *p*1 = 0.028, *ω*1 = 1.000; *p*2 = 0.001, *ω*2 = 6.668; *lnL* = −9,060.276	*2∆lnL* = 3.581, *p* =.167
*Vg‐like‐C*	*p*0 = 0.877, *ω*0 = 0.075; *p*1 = 0.123, *ω*1 = 1.000; *lnL* = −2,892.983	*p*0 = 0.886, *ω*0 = 0.078; *p*1 = 0.000, *ω*1 = 1.000; *p*2 = 0.114, *ω*2 = 1.071; *lnL* = −2,892.958	*2∆lnL* = 0.050, *p* =.975

The branch‐site models as well as Benjamini–Hochberg correction (*n* = 10) showed that for nine of the ten *Bombus* subgenera (except for *B*. *Psithyrus)*, three or more sites were under positive selection with posterior probability >0.5. And in four subgenera (*Pyrobombus*, *Sibiricobombus*, *Melanobombus*, and *Alpigenobombus*), at least one site was under positive selection with posterior probability >.95. For the same nine subgenera, the log‐likelihood values of Model A significantly deviated from its counterpart Model A1 (*p* =< .016), indicating the likelihood of this result. For *Psithyrus*, only one site showed signs of positive selection. However, because there were not any deviations between Model A and Model A1 (*2∆lnL* = 0.000, *p* = 1.000), this result could not be supported. The details of the branch‐site model results are listed in Table 6.

**TABLE 6:** Branch‐site model results for each of the 10 bumblebee subgenera (*N*_0.5_, number of sites which were positively selected with posterior probability >0.5; *N*_0.95_, number of sites which were positively selected with posterior probability >0.95)

Foreground subgenus	LRT (*df* = 1)	*N*_0.5_	*N*_0.95_
*Pyrobombus*	*2∆lnL* = 94.471, *p* < .001	52	10
*Bombus* *s. str*.	*2∆lnL* = 6.709, *p* = .012	3	0
*Sibiricobombus*	*2∆lnL* = 12.667, *p* < .001	26	1
*Melanobombus*	*2∆lnL* = 57.192, *p* < .001	80	5
*Alpigenobombus*	*2∆lnL* = 19.542, *p* < .001	41	1
*Thoracobombus*	*2∆lnL* = 27.831, *p* < .001	107	0
*Psithyrus*	*2∆lnL* = 0.000, *p* = 1.000	1	0
*Megabombus*	*2∆lnL* = 10.140, *p* = .002	15	0
*Subterraneobombus*	*2∆lnL* = 6.037, *p* = .016	6	0
*Mendacibombus*	*2∆lnL* = 41.450, *p* < .001	44	0

## 4 DISCUSSION

Vitellogenin is a multifunctional hemolymph protein that is characterized by its central role in social insect's division of labor and affects multiple aspects of social insect life histories (Amdam et al., 2003). Bumble bees are primitively eusocial insects which represents an intermediate stage in the evolution between solitary and eusociality (Goulson, 2010). Unlike the “true” eusocial bees such as honey bees or stingless bees, most bumble bee species pass through both solitary and eusocial phases during their life cycle. Moreover, bumble bees are extremely diversified in their biological and ecological characteristics among different subgenera. Here we show that in the bumble bees, *Vg* has experienced elevated rate of evolution and is under strong positive selection. Its homologous genes, *Vg‐like* genes that have diverged both from *Vg* and from each other, do not display such patterns. Additionally, signs of positive selection were also absent from its sister clades, *Tetragonula* and *Apis's* *Vg*.

A unique advantage of bumble bees is the fact that sequences from the two closest clades are publicly available, so we can investigate the forces driving vitellogenin evolution across the phylogeny tree and ask whether *Vg* and *Vg‐like* genes in distinctive genus differ in their molecular evolutionary rates. Our study determines that the conventional Vg is the most rapidly evolving copy in bumblebees. Interestingly, we observed that unlike in bumble bees, no sign of positive selection could be found for the conventional *Vg* of both *Tetragonula* and *Apis*. The pairwise analyses showed that all the three genera had increased *dN* with increasing *dS*. However, the slope of the linear regression of *Bombus* was much higher than that of *Tetragonula* and *Apis*, indicating a higher overall *dN/dS* ratio in *Bombus*. Although there were considerable overlaps of *dS* between *Bombus* and the other two genera, the pairwise *dN/dS* values among *Bombus* genera were larger than any of the counterparts of *Tetragonula* and *Apis* (Figure 2). In *Tetragonula* and *Apis*, the purifying selection pressure on *Vg* might be slightly lower because *Vg‐*related traits underlying adaptive evolution may differ between the genera. Thus, our results suggest that duplication, positive evolution, and purifying selection may be major evolutionary forces driving Vg gene evolution across divergent taxa.

Vg is best known for its primary role in the formation of egg yolk in egg‐laying animals (Tufail & Takeda, 2008); however, in social insects, Vg has probably acquired additional functions (Guidugli et al., 2005) and fulfills roles related to behavior and survival (Havukainen et al., 2011; Kent et al., 2011; Nelson et al., 2007; Seehuus et al., 2006). Functional pleiotropy plays an important role in molecular evolution (Paaby & Rockman, 2013). Although increased purifying selection against pleiotropic mutations (McGuigan et al., 2014) and slow rates of evolution of pleiotropic genes (Salathe et al., 2006) have been observed in some cases, more recent studies have demonstrated that gene pleiotropy may increase evolutionary rate (Razeto‐Barry et al., 2011; Twyman et al., 2018; Vedanayagam & Garrigan, 2015). While viewed as a primitively eusocial genus, the single conventional Vg protein in bumble bees showed inclines of pleiotropy in previous studies. For instance, *Vg* was found to be expressed in *B*. *hypocrita* in several castes including queen, worker, and even drone (Li et al. 2010). Additionally, *Vg* mRNA was detected in various tissues including flight muscles in *B*. *terrestris* as well as *B*. *lantschouensis* (Jedlicka et al., 2016; Zhen et al., 2018). This pattern is consistent with the fact that the positive selection that we detected may drive the evolution of novel protein function and thus enables functional pleiotropy of bumble bee's Vg (Razeto‐Barry et al., 2011). Similar patterns have been observed in the *Vg* genes of other eusocial hymenopteran species (Kent et al., 2011; Morandin et al., 2014; Salmela et al., 2016). Thus, our results suggest that Vg functional pleiotropy may have arisen due to strong positive selection acting on it, and may further indicate the appearance of a novel, unknown Vg function in bumble bees.

Functional pleiotropy of a gene is also predicted to lead to a duplication event, such as the duplication of the ancestral gene leading to *Vg* and *Vg‐like* genes (Morandin et al., 2014). Gene duplication is an important source of new genetic material for selection to act upon (Force et al., 1999; Lynch & Force, 2000; Ohno, 1970; Zhang, 2003). After duplication, the duplicated gene copy can acquire functions different from those of the ancestral gene (Gu et al., 2002; Khaladkar & Hannenhalli, 2012; Morandin et al., 2014; Wagner, 2000). Unlike *Vg*, the three *Vg‐like* genes showed signs of purifying selection in all three genera. Multiple factors could be affecting the rate of sequence evolution such as the number of pleiotropic interactions, the gene expression levels, or their tissue‐specific expression patterns, that could not be detected in this study. Further studies on evolutionary patterns of *Vg‐like* genes across social insect species and on their functions are needed to fully understand their roles in social insects and the selection pressures they experience.

An exception to the otherwise overall positive selection on vitellogenin in our set of bumble bee species is the subgenus *Psithyrus*. We found that the subgenus‐based branch model analyses showed that almost all subgenera had a *dN/dS* ratio over 1. Incredibly however, the *dN/dS* ratio of the subgenus *Psithyrus* was much lower than the other subgenera (only 0.713). Moreover, the subgenus‐based branch‐site model analyses indicated that all subgenera but *Psithyrus* had significant deviations between Model A (alternative hypothesis) and Model A1 (null hypothesis). These results repeatedly indicate the distinctive evolution of *Psithyrus Vg*. Bumble bees in the subgenus *Psithyrus* have annual life cycles similar to those of typical bumble bee species, except that instead of founding their own nest and rearing workers, they steal a nest from “true” bumble bees (Goulson, 2010). Initially, these bumble bees were formerly described as a separate genus *Psithyrus*, but it is now widely accepted that they belong to a subgenus within *Bombus*, with the subgenus *Thoracobombus* as the sister group (Cameron et al., 2007; Williams et al., 2008). These bumblebees also exhibit social parasitism, with the absence of a worker caste, or the need to forage for nectar and pollen to provision developing larvae (Lhomme & Hines, 2019). Although it is not clear whether the lower positive selection level of *Psithyrus* was a secondary evolutionary event, it is probable that their fundamentally different life history has influenced the evolution of *Vg* even within just one genus. In fact, due to the absence of caste differentiation in *Psithyrus*, it is conceivable that its simplified lifestyle reduced the functional pleiotropy demand on *Vg* and thus caused a lower *dN/dS* ratio.

## CONFLICT OF INTEREST

The authors declare no conflict of interest.

## AUTHOR CONTRIBUTION

**Fang Zhao:** Investigation (equal); Methodology (equal). **Claire Morandin:** Investigation (equal); Methodology (equal). **Kai Jiang:** Methodology (supporting). **Tianjuan Su:** Methodology (supporting). **Bo He:** Methodology (supporting). **Gonghua Lin:** Conceptualization (lead); Funding acquisition (equal); Investigation (lead); Methodology (equal). **Zuhao Huang:** Funding acquisition (equal); Investigation (equal); Methodology (equal); Project administration (lead).

## Supporting information

SupInfo S1

SupInfo S2

## Data Availability

The genome data (BioProject ID: PRJNA667279) are available from the Sequence Read Archive of the National Centre for Biotechnology Information (NCBI). The other data generated or analyzed during this study are included in this published article and its supplementary files.
