# Supplementary material for: Molecular evolution of bumble bee vitellogenin and vitellogenin‐like genes
Source: Ecol Evol. 2021 Jun 5;11(13):8983–92. doi: 10.1002/ece3.7736 (PMC8258195; doi:10.1002/ece3.7736)
Supplement: Supplementary file 2 — SupInfo S2 [file ECE3-11-8983-s002.docx]

**Table S1.** Bumblebee species involved in this study.

| No. | Species | Subgenus | GenBank ID | References |
| --- | --- | --- | --- | --- |
| S01 | *B. vancouverensis* | *Pyrobombus* (Pr) | GCA_011952275.1 | Jackson et al 2020 |
| S02 | *B. vosnesenskii* | *Pyrobombus* (Pr) | GCF_011952255.1 | Jackson et al 2020 |
| S03 | *B. impatiens* | *Pyrobombus* (Pr) | GCF_000471645.3 | Sadd et al 2015 |
| S04 | *B. melanopygus* | *Pyrobombus* (Pr) | SRR8700092 | Tian et al 2019 |
| S05 | *B. lepidus* | *Pyrobombus* (Pr) | SRR8283904 | Lin et al 2019 |
| S06 | *B. lantschouensis* | *Bombus s. str.* (Bo) | SRR12770617 | this study |
| S07 | *B. terricola* | *Bombus s. str.* (Bo) | SRR7696608 | Kent et al 2018 |
| S08 | *B. lucorum* | *Bombus s. str.* (Bo) | SRR8283905 | Lin et al 2019 |
| S09 | *B. terrestris* | *Bombus s. str.* (Bo) | GCF_000214255.1 | Sadd et al 2015 |
| S10 | *B. asiaticus* | *Sibiricobombus* (Sb) | SRR8283910 | Lin et al 2019 |
| S11 | *B. sibiricus* | *Sibiricobombus* (Sb) | SRR8283899 | Lin et al 2019 |
| S12 | *B. ladakhensis* | *Melanobombus* (ML) | SRR8283908 | Lin et al 2019 |
| S13 | *B. pyrosoma* | *Melanobombus* (ML) | SRR8283909 | Lin et al 2019 |
| S14 | *B. sichelii* | *Melanobombus* (ML) | SRR12770616 | this study |
| S15 | *B. kashmirensis* | *Alpigenobombus* (AL) | SRR8283906 | Lin et al 2019 |
| S16 | *B. breviceps* | *Alpigenobombus* (AL) | SRR8283907 | Lin et al 2019 |
| S17 | *B. impetuosus* | *Thoracobombus* (Th) | SRR12770615 | this study |
| S18 | *B. laesus* | *Thoracobombus* (Th) | SRR12770614 | this study |
| S19 | *B. skorikovi* | *Psithyrus* (Ps) | SRR12770613 | this study |
| S20 | *B. bohemicus* | *Psithyrus* (Ps) | SRR12770612 | this study |
| S21 | *B. supremus* | *Megabombus* (Mg) | SRR8283902 | Lin et al 2019 |
| S22 | *B. bicoloratus* | *Megabombus* (Mg) | SRR8283903 | Lin et al 2019 |
| S23 | *B. trifasciatus* | *Megabombus* (Mg) | SRR12770611 | this study |
| S24 | *B. melanurus* | *Subterraneobombus* (St) | SRR8283901 | Lin et al 2019 |
| S25 | *B. personatus* | *Subterraneobombus* (St) | SRR8283900 | Lin et al 2019 |
| S26 | *B. waltoni* | *Mendacibombus* (Md) | SRR12770610 | this study |
| S27 | *B. convexus* | *Mendacibombus* (Md) | SRR12770609 | this study |

**References:**

Jackson JM, Pimsler ML, Oyen KJ, Strange JP, Dillon ME, Lozier JD. Local adaptation across a complex bioclimatic landscape in two montane bumble bee species. Molecular Ecology, 2020, 29: 920–939.

Kent CF, Dey A, Patel H, Tsvetkov N, Tiwari T, MacPhail VJ, Gobeil Y, Harpur BA, Gurtowski J, Schatz MC, Colla SR, Zayed A. Conservation Genomics of the Declining North American Bumblebee Bombus terricola Reveals Inbreeding and Selection on Immune Genes. Frontiers in Genetics, 2018, 9:316

Lin G, Huang Z, Wang L, Chen Z, Zhang T, Gillman LN, Zhao F. Evolutionary rates of bumblebee genomes are faster at lower elevations. Molecular Biology and Evolution, 2019, 36(6): 1215–1219.

Sadd BM, Barribeau S, Bloch G, de Graaf DC, Dearden P, Elsik CG, et al. The genomes of two key bumblebee species with primitive eusocial organization. Genome Biology, 2015, 16:76.

Tian L, Rahman SR, Ezray BD, Franzini L, Strange JP, Lhomme P, Hines HM. A homeotic shift late in development drives mimetic color variation in a bumble bee. PNAS, 2019, 116 (24): 11857-11865.

**Table S2.** *Tetragonula* and *Apis* species involved in this study.

| Species | GenBank accession | References |
| --- | --- | --- |
| *Tetragonula_carbonaria* | GCA_010645115.1 | Hereward et al. 2020 |
| *Tetragonula clypearis* | GCA_010645135.1 | Hereward et al. 2020 |
| *Tetragonula davenporti* | GCA_010645165.1 | Hereward et al. 2020 |
| *Tetragonula hockingsi* | GCA_010645185.1 | Hereward et al. 2020 |
| *Tetragonula_mellipes* | GCA_011634685.1 | Hereward et al. 2020 |
| *Apis cerana* | GCF_001442555.1 | Park et al. 2015 |
| *Apis dorsata* | GCF_000469605.1 | Oppenheim et al. 2020 |
| *Apis florea* | GCF_000184785.3 | Qu et al. 2019 |
| *Apis mellifera* | GCF_000002195.4 | Wallberg et al. 2019 |
| *Apis laboriosa* | GCA_014066325.1 | Lin et al. 2020 |

**References:**

Hereward JP, Smith TJ, Brookes DR, Gloag R, Walter GH. Tests of hybridisation in Tetragonula stingless bees using multiple genetic markers. bioRxiv preprint doi: 10.1101/2020.03.08.982546.

Park D, Jung JW, Choi BS, Jayakodi M, Lee J, Lim J, Yu Y, Choi YS, Lee ML, Park Y, Choi IY, Yang TJ, Edwards OR, Nah G, Kwon HW. Uncovering the novel characteristics of Asian honey bee, Apis cerana, by whole genome sequencing. BMC Genomics. 2015, 16(1):1.

Wallberg A, Bunikis I, Pettersson OV, Mosbech MB, Childers AK, Evans JD, Mikheyev AS, Robertson HM, Robinson GE, Webster MT. A hybrid de novo genome assembly of the honeybee, Apis mellifera, with chromosome-length scaffolds. BMC Genomics. 2019 Apr 8;20(1):275.

Oppenheim S, Cao X, Rueppel O, Krongdang S, Phokasem P, DeSalle R, Goodwin S, Xing J, Chantawannakul P, Rosenfeld JA. Whole Genome Sequencing and Assembly of the Asian Honey Bee Apis dorsata. Genome Biol Evol. 2020 Jan 1;12(1):3677-3683.

Lin D, Lan L, Zheng T, Shi P, Xu J, Li J. Comparative genome analysis reveals the adaptive evolution of the Himalayan giant honeybee Apis laboriosa. (Unpublished, Submitted on 22-JUL-2020). <https://www.ncbi.nlm.nih.gov/nuccore/JACEOM000000000.1/>

Qu J, Richards S, Aqrawi P, Blankenburg K, et al. Direct Submission. (Unpublished, Contigs were updated on November 2019). <https://www.ncbi.nlm.nih.gov/nuccore/AEKZ00000000.1/>

**Table S3.** Positively selected sites (M2a model) of bumblebee *Vg* and *Vg-like* genes

| Gene | Positively selected sites (with posterior probability >0.95) |
| --- | --- |
| *Vg* | 27T, 35L, 36T, 39D, 70H, 71T, 106V, 151M, 159Q, 186S, 189I, 192I, 234R, 235Q, 276H, 299N, 312T, 362L, 373H, 397G, 418S, 430M, 441C, 442H, 466L, 508N, 538G, 545P, 546A, 569M, 572Q, 585H, 592Y, 632F, 641R, 660S, 679D, 711W, 731D, 736T, 743A, 750P, 762V, 767T, 769E, 805F, 832G, 850E, 858L, 865G, 869F, 878L, 887L, 891R, 896R, 897L, 898G, 920L, 922L, 929D, 934V, 936H, 937P, 939A, 941K, 942A, 992P, 994G, 996F, 1027D, 1039I, 1047S, 1050T, 1051A, 1056V, 1058V, 1059D, 1062V, 1064N, 1066D, 1075F, 1077V, 1079T, 1094L, 1102V, 1107F, 1113S, 1119N, 1121E, 1123V, 1133T, 1151D, 1168A, 1169V, 1173L, 1180G, 1203S, 1204L, 1205E, 1206T, 1213T, 1247Y, 1269R, 1273L, 1307S, 1310V, 1316S, 1318T, 1320Y, 1328V, 1341R, 1343E, 1345L, 1349M, 1357M, 1366R, 1369L, 1370L, 1379H, 1388L, 1392A, 1449R, 1460R, 1462Y, 1464L, 1498L, 1499P, 1509K, 1515S, 1530Q, 1532G, 1534Y, 1554L, 1593L, 1604R, 1609F, 1617S, 1621Y, 1625N, 1639R, 1652V, 1656Q, 1670T, 1677Q, 1686W, 1692S, 1704A, 1705T, 1708A, 1728L |
| *VgA* | 243S, 1318T, 1512T |
| *VgB* | — |
| *VgC* | — |


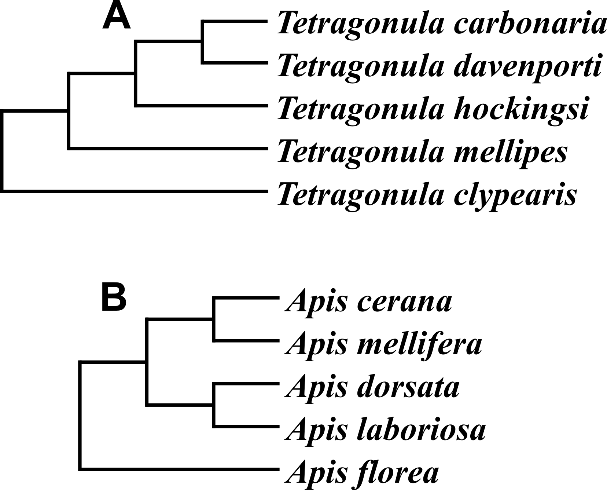


**Figure S1.** Phylogenetic relationships among *Tetragonula* species (A) and *Apis* species (B) involved in this study. The Tetragonula tree was drawn according to Rasmussen and Cameron (2010) and Hereward et al. (2020) while the Apis tree was also drawn according to Raffiudin and Crozier (2007).

**References:**

Rasmussen C and Cameron SA. Global stingless bee phylogeny supports ancient divergence, vicariance, and long distance dispersal. Biological Journal of the Linnean Society, 2010, 99, 206–232.

Hereward JP, Smith TJ, Brookes DR, Gloag R, Walter GH. Tests of hybridisation in Tetragonula stingless bees using multiple genetic markers. bioRxiv preprint doi: 10.1101/2020.03.08.982546.

Raffiudin R, Crozier RH. Phylogenetic analysis of honey bee behavioral evolution. Molecular Phylogenetics and Evolution 43 (2007) 543–552.


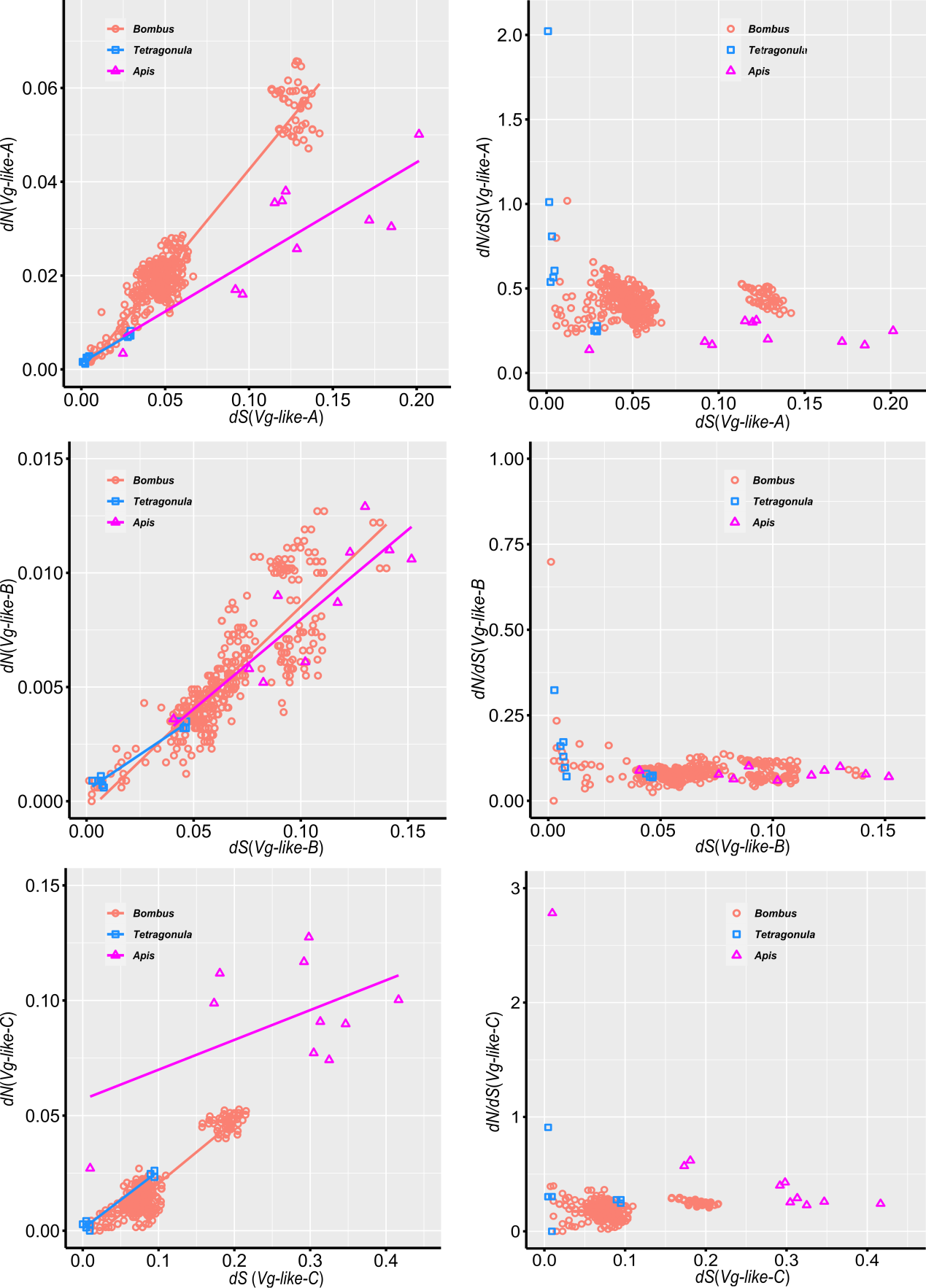


**Figure S2.** Scatter plot of *dN* vs. *dS* (left) and *dN/dS* vs. *dS* (right) in *Vg-like* genes. The *dN*, *dS*, and *dN/dS* values were calculated under pairwise models among different species within genus *Bombus*, *Tetragonula*, and *Apis*, respectively.
